# Therapeutic Use of Cerebellar Intermittent Theta Burst Stimulation (iTBS) in a Sardinian Family Affected by Spinocerebellar Ataxia 38 (SCA 38)

**DOI:** 10.1007/s12311-021-01313-z

**Published:** 2021-08-19

**Authors:** Angela Sanna, Paolo Follesa, Paolo Tacconi, Mariangela Serra, Maria Giuseppina Pisu, Viola Cocco, Michela Figorilli, Giovanni Defazio, Monica Puligheddu

**Affiliations:** 1 Department of Biomedical Sciences, University of Cagliari, Cagliari, Italy; 2 Department of Life and Environment Sciences, Section of Neuroscience and Anthropology and Center of Excellence for Neurobiology of Dependence, University of Cagliari, Cagliari, Italy; 3 Section of Neurology, University Hospital of Cagliari, Cagliari, Italy; 4 Neuroscience Institute, National Research Council of Italy (CNR), Cagliari, Italy; 5 Department of Medical Science and Public Health, Section of Neurology, University of Cagliari, Cagliari, Italy

**Keywords:** Cerebellar ataxia, SCA 38, Theta burst stimulation, Transcranial magnetic stimulation, Brain-derived neurotrophic factor

## Abstract

Spinocerebellar ataxia 38 (SCA 38) is an autosomal dominant disorder caused by conventional mutations in the ELOVL5 gene which encodes an enzyme involved in the synthesis of very long fatty acids, with a specific expression in cerebellar Purkinje cells. Three Italian families carrying the mutation, one of which is of Sardinian descent, have been identified and characterized. One session of cerebellar intermittent theta burst stimulation (iTBS) was applied to 6 affected members of the Sardinian family to probe motor cortex excitability measured by motor-evoked potentials (MEPs). Afterwards, patients were exposed to ten sessions of cerebellar real and sham iTBS in a cross-over study and clinical symptoms were evaluated before and after treatment by Modified International Cooperative Ataxia Rating Scale (MICARS). Moreover, serum BDNF levels were evaluated before and after real and sham cerebellar iTBS and the role of *BDNF* Val66Met polymorphism in influencing iTBS effect was explored. Present data show that one session of cerebellar iTBS was able to increase MEPs in all tested patients, suggesting an enhancement of the cerebello-thalamo-cortical pathway in SCA 38. MICARS scores were reduced after ten sessions of real cerebellar iTBS showing an improvement in clinical symptoms. Finally, although serum BDNF levels were not affected by cerebellar iTBS when considering all samples, segregating for genotype a difference was found between Val66Val and Val66Met carriers. These preliminary data suggest a potential therapeutic use of cerebellar iTBS in improving motor symptoms of SCA38.

## Introduction

Spinocerebellar ataxias (SCAs) are rare autosomal dominant neurological disorders characterized by progressive cerebellar ataxia, resulting in unsteady gait, clumsiness, and dysarthria. Atrophies of the cerebellum and brainstem are most often the prominent feature, but other structures can be affected, leading to several phenotypes [1]. Genetically, they are grouped as repeated expansion SCAs and conventional mutations SCAs. The latter are generally less severe and show a slower disease progression [1, 2]. SCA 38 is an autosomal dominant disorder caused by conventional mutations in the *ELOVL5* gene which encodes an enzyme involved in the synthesis of very long fatty acids, with a specific expression in cerebellar Purkinje cells [3]; docosahexanoid acid (DHA) supplementation has been proved to be effective for improving clinical features and brain metabolism at short- and long-term follow up [4–6]. The disease is very rare: one French, one Spanish, and three Italian affected families, one of which is a Sardinian kindred, have been identified and characterized so far [6, 7]. Brain MRI showed selective cerebellar atrophy with normal brainstem and cerebral cortex; the disease is slowly progressive with onset at 39.1 years of median age (range 26–50) and no sex differences. Initial clinical features are gait ataxia associated with hyposmia and pes cavus, followed by limb ataxia, dysarthria, dysphagia, ophtalmoparesis, and sensory loss. By the fourth decade of the disease, patients are wheelchair-bound and dependent in daily basic activities. The mean disease duration from onset to death is 41 years. Patients do not display cognitive impairment, while anxiety disorder and hearing loss have been described in the Sardinian family [7].

Non-invasive brain stimulation (NIBS) techniques have been applied to the cerebellum of patients affected by different forms of ataxias for diagnostic and therapeutic purposes [8–11] showing encouraging results. The mechanism underlying the effect of cerebellar stimulation is still an object of debate and may involve the modulation of the cerebellar-thalamo-cortical pathway as well as other complex mechanisms such as induction of gene expression, regulation of neurotransmitter activity, and influence on signal transduction pathways [10–12]. Theta burst stimulation (TBS) is a patterned protocol of repetitive transcranial magnetic stimulation (rTMS) which can be delivered in an excitatory (intermittent TBS) or inhibitory (continuous TBS) fashion [13] and can produce long-lasting effects on cortical excitability through long-term potentiation and depression (LTP and LTD) mechanisms [14, 15]. TBS is considered a promising therapeutic tool because it requires low stimulus intensities and short stimulation times, but, as with other TMS protocols, its routine use is limited by a high intra- and inter-subject subject variability of response, often requiring a large number of patients to obtain a statistical significant response [16, 17]. Many factors such as genetic polymorphisms, age, sex, cortical activity, synaptic activation, and technical factors may affect the response to TBS [14, 16]; among these, a pivotal role is played by a common polymorphism of the *BDNF* gene, Val66Met, which has been associated with the onset and progression of several neurodegenerative disorders; however data are conflicting [18–21]. Moreover, the altered synaptic plasticity associated with the presence of the polymorphism may induce changes in cortical excitability [22, 23] and a lack of response to TMS treatment and especially to TBS [14, 24–26]. Likewise, BDNF serum levels have been described as a possible biomarker of TMS and TBS after-effect but published data are controversial [27–30]. Indeed, it has been shown that LTP and LTD protocols can respectively increase and decrease BDNF serum levels, but other studies found an opposite trend or no effect [27–30].

Based on this evidence, the aim of this study is to investigate the efficacy of 2 weeks of cerebellar intermittent TBS (iTBS) applied to the cerebellum, in improving clinical signs of SCA-38. The study also explores changes in serum BDNF levels following iTBS treatment and the possible involvement of Val66Met polymorphism in influencing response to iTBS.

## Patients and Methods

### Patients

Six patients previously diagnosed with SCA-38 [3], belonging to the same Sardinian family, were enrolled. Two other carriers of the mutation in the same family did not give their consent to the treatment. Patients underwent a complete neurological examination and Modified International Cooperative Ataxia Rating Scale (MICARS) evaluation [31].

Inclusion criteria were age ≤ 80 and > 18 years and diagnosis of SCA 38 with the presence of clinical symptoms. Exclusion criteria were inability to understand and sign the informed consent, other severe neurological disorders, significant medical or psychiatric illnesses, history of epilepsy or seizures, and pregnancy.

All experimental procedures were approved by the local Ethical Committee (CEI Azienda Ospedaliero-Universitaria di Cagliari PG/2018/8829. Study Code: TMS-BIOMK). All patients gave written consent for the study and data publication. The study endorsed the Principles of Human Rights, as adopted by the World Medical Association (18th WMA General Assembly) in 1964 in Helsinki (Finland) and then amended by the 64th WMA General Assembly in 2013 in Fortaleza (Brazil).

### Experimental Design

**Fig. 1:**
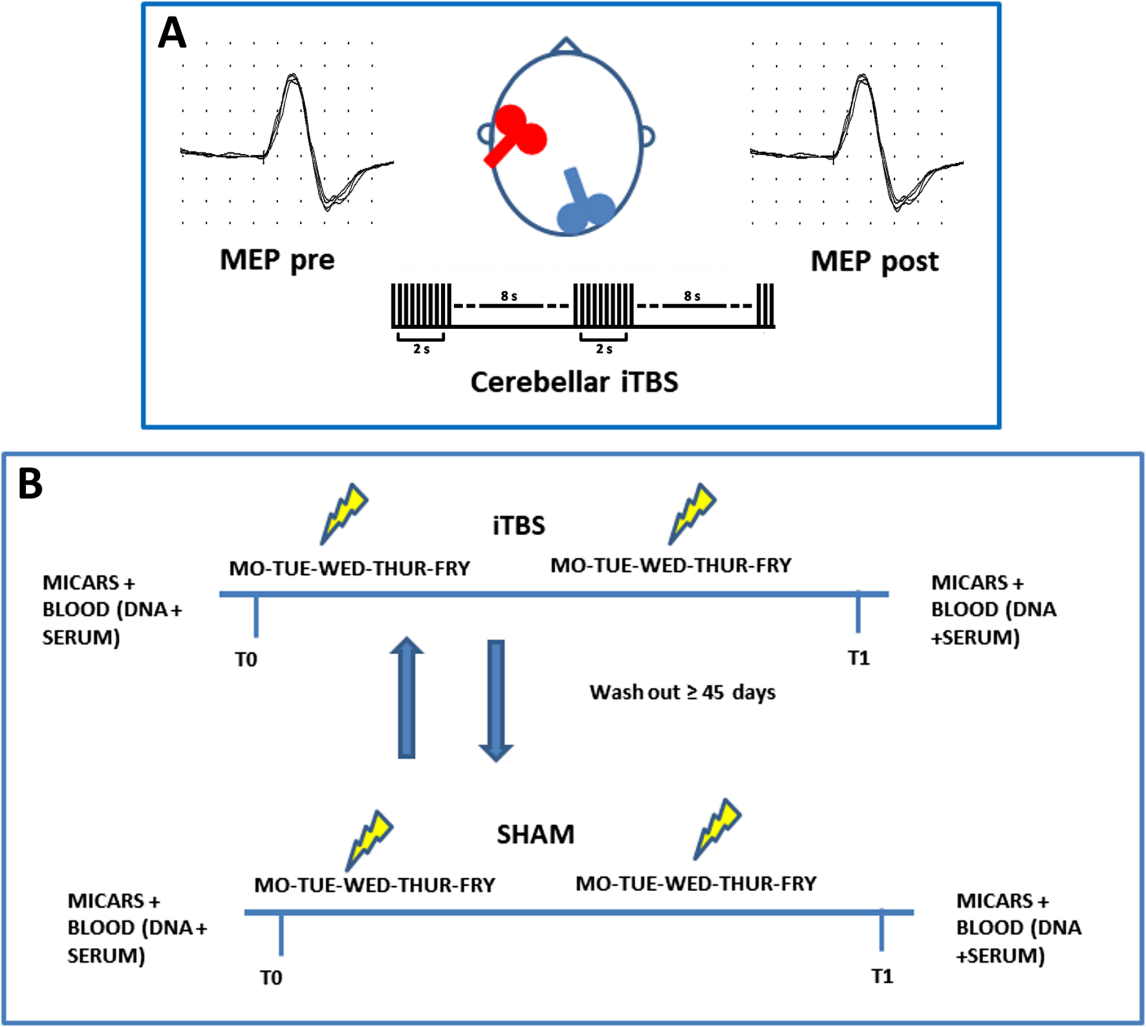
Experimental design. **A** The procedure to assess the effect of one session of cerebellar stimulation on MEP. **B** The procedure of the chronic sham controlled cross over cerebellar iTBS treatment

Motor cortex excitability was evaluated before and after one session of cerebellar iTBS. Thereafter, a chronic double-blind sham-controlled cross-over treatment was performed (Fig. 1). Patients were randomized to receive 10 sessions of real and 10 sessions of sham iTBS, separated by 45 days. Clinical evaluation was made by MICARS at the beginning and end of each trial; patients were video-recorded, and two blinded evaluators generated MICARS scores. Blood samples were collected at the beginning and end of each trial. Before clinical assessment, nerve conduction studies were performed to evaluate the presence of polyneuropathy.

### Nerve Conduction Studies

Nicolet Viking EDX (Natus Inc., Pleasanton, CA) was used to perform nerve conduction studies with standard methods (for details, see [32]). Briefly, motor nerve conduction was studied stimulating fibular and ulnar nerves at the ankle, knee, wrist, and elbow; compound muscle action potentials (CMAPs) were recorded with surface electrodes from abductor digiti minimi and extensor digitorum brevis. Antidromic sensory nerve conduction was studied stimulating sural and superficial radial nerves and recording sensory nerve action potentials (SNAPs) with surface electrodes placed behind the malleolus and at the wrist. Moreover, H-reflex was recorded from soleus muscle stimulating the tibial nerve in the popliteal fossa.

### Intermittent Theta Burst Stimulation

a Magstim super rapid ^2^ stimulator (The Magstim Company Ltd., Whitland, UK) connected to a figure of eight air-cooled coil was employed. The targeting sites of the cerebellum were 1 cm inferior and 3 cm left/right to the inion, and the coil was positioned tangentially to the scalp, with the handle pointing superiorly, as previously described [12]. The iTBS consisted of 20 cycles of 2 s of three-pulse bursts at 50 Hz repeated every 200 ms (5 Hz) repeated every 10 s for a total of 600 pulses [13, 33]. Stimulator intensity was set at 80% of the active motor threshold (AMT) of the first dorsal interosseous. A 2-min pause was set between left/right stimulation. Sham stimulation was performed with the coil angled at 90° to the skull decreasing the power to 40% of AMT.

### Effects of iTBS of the Lateral Cerebellum on MEPs

Subjects were asked to relax and keep their eyes open. Single-pulse TMS was delivered through a Magstim super rapid^2^ stimulator (The Magstim Company Ltd., Whitland, UK) connected to a figure of eight coil placed over the left M1 in the optimal position for eliciting MEPs in the right FDI muscle. Twenty single pulses delivered at the intensity able to evoke 1-mV MEPs were collected before and 10 min after iTBS applied to the lateral right cerebellum [12].

### Genotyping

Genomic DNA was extracted from blood using standard procedures and a commercial kit (Sigma-Aldrich, Milan, Italy) and then used as template to detect specific target containing the rs6265 BDNF polymorphism (GenBank accession number: AB038670). Genomic DNA was amplified by polymerase chain reaction (PCR) with primers (forward 5′-AAAGAAGCAAACATCCGAGGACAAG-3′; reverse 5′-GGAGAAGAGAAAGACGACCTCCTTA-3′) generating the 273-bp amplicon that includes the BDNF Val66Met detectable by Hin1 II digestion as previously described with slight modifications [29]. Briefly, DNA was amplified by PCR using 0.4 μM of primers, 20 ng of genomic DNA in 75 mM Tris–HCl (pH 9.0), 20 mM ammonium sulphate, 0.01% Tween 20, 1.5 mM magnesium chloride, 0.4 mM dNTP, and 1 U Taq polymerase. The specific PCR protocol used was as follows: 5 min at 95 °C, 35 cycles at 95 °C for 30 s, 55 °C for 40 s, 72 °C for 50. The 35 cycles were followed by a final extension at 72 °C for 5 min. PCR products were digested with Hin1 II restriction enzyme to detect the G → A SNP.

### Serum BDNF

Serum-collecting tubes were used to take blood samples before (T0) and at the end (T90) of each session. Serum was obtained by centrifugation of collecting tubes at 900 × g for 10 min. The resulting supernatant was frozen at − 80 °C until use for BDNF assay. A standard enzyme-linked immunosorbent assay (ELISA) commercial kit (Sigma-Aldrich, Milan, Italy) was used to measure the amount of BDNF in each sample as previously described [29, 34]. ELISA was performed according to the manufacturer’s instruction using a 96-well plate that was pre-coated with a primary antibody against rat BDNF. Each sample was run in duplicate.

### Statistical Analysis

GraphPad Prism 8.01 software (San Diego, CA, USA) was used for statistical analysis. The Shapiro–Wilk test was used to assess normality in data distribution. Parametric *t*-test was used to evaluate differences in MEP amplitude. The non-parametric Wilcoxon test was applied to the mean MICARS total, and then the subscales were considered separately applying Wilcoxon test to each sub-scale, as previously described [35].

## Results

### Patients

Table 1 shows demographic features, MICARS score at baseline, and BDNF genotype.

**Table 1:** Demographic, clinical and genetic features of SCA 38 patients. AMT: activated motor threshold expressed as percentage of maximum stimulator output

Patient #	Age	Sex	Age at diagnosis	MICARS score	AMT	Polyneuropathy	BDNF genotype
1	56	F	47	30	50	No	Val/Val
2	45	M	34	21	45	No	Val/Val
3	54	M	45	18	50	Yes	Val/Val
4	53	M	43	15	70	No	Val/Met
5	50	F	41	11	40	No	Val/Met
6	44	F	35	24	45	No	Val/Met

### Nerve Conduction Studies

Among 6 patients, only one had mild sensory-motor axonal neuropathy. The remaining patients had normal conduction velocities, CMAP, and SNAP amplitudes.

### Effect of a Single Session of Cerebellar iTBS on MEPs

**Fig. 2:**
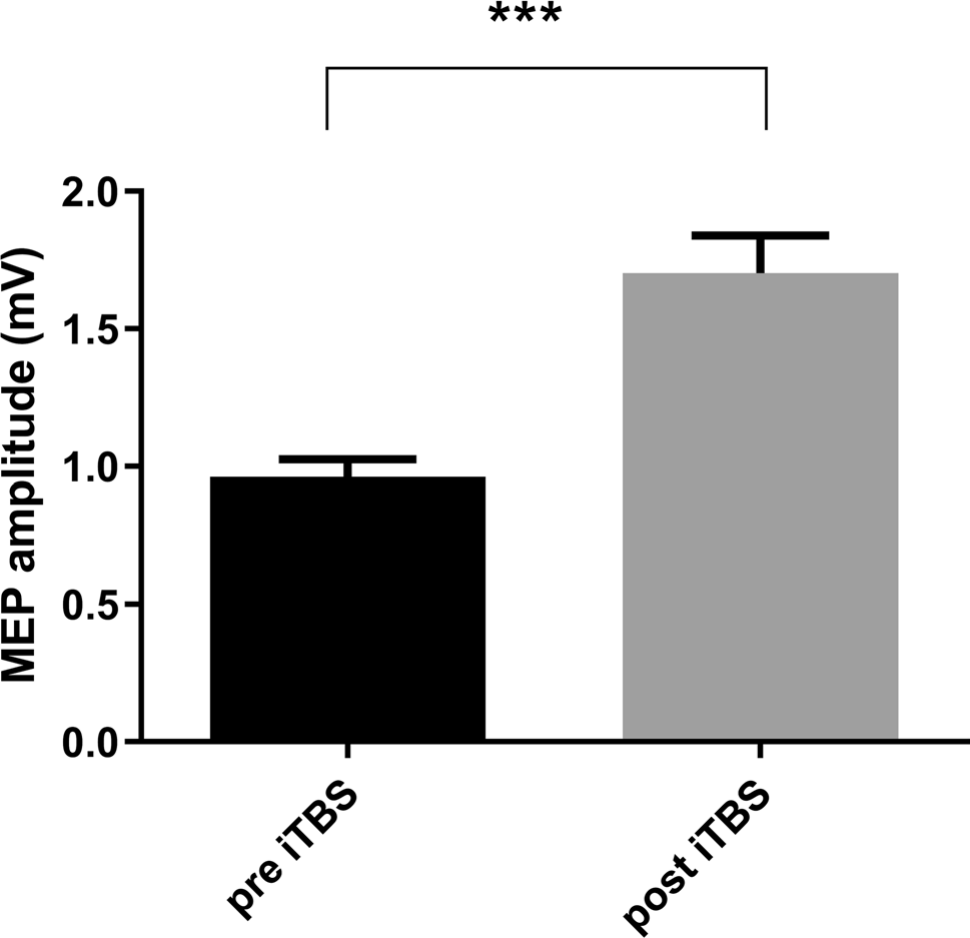
Effect of one session of cerebellar iTBS on MEP amplitude. Data are normalized and presented as mean ± SEM. ***p < 0.001 post vs pre-iTBS

Figure 2 shows a significant increase of MEP amplitude 10 min after cerebellar iTBS (1.0 ± 0.03 vs 1.7 ± 0.13; *p* = 0.0005, effect size > 1.0). Segregating for *BDNF* genotype, no difference was found between Val66Val and Val66Met patients (*p* ˃ 0.05).

### Effect of 10 Sessions of Cerebellar iTBS on Motor Symptoms

**Fig. 3:**
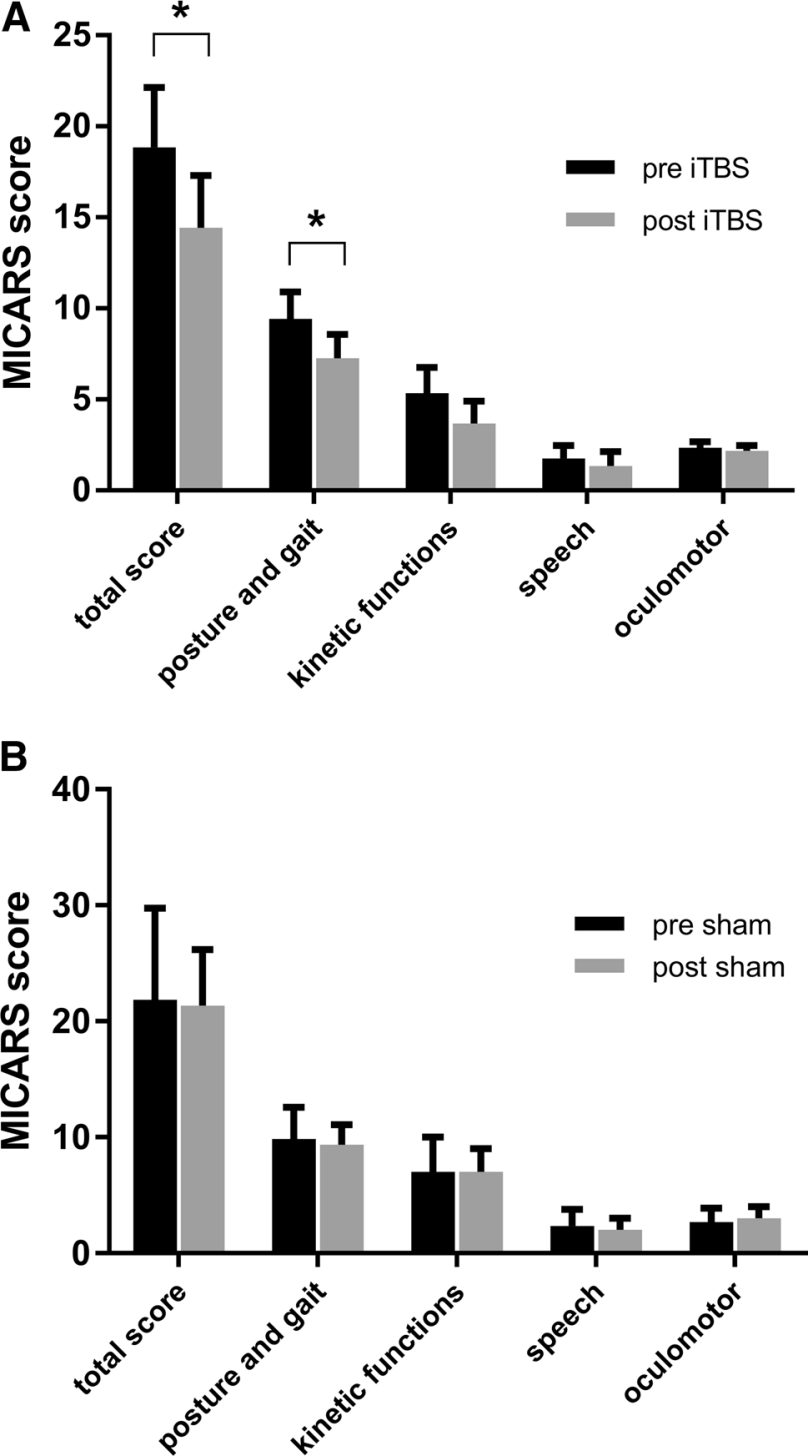
MICARS total and subscale scores obtained by patients before and after 10 sessions of cerebellar iTBS (**A**) and Sham stimulation. Data are presented as mean ± SEM. *p < 0.05 post vs pre-iTBS

Figure 3 shows a significant decrease in MICARS score after real iTBS applied to the cerebellum. The total and subscale scores were tested pre-TBS and post-TBS. Mean MICARS total scores decreased after iTBS treatment (18.83 ± 3.3 vs 14.42 ± 2.9 *p* = 0.03, effect size > 0.5). Considering the subscales, only the posture and gait disturbance section displayed a significant decrease (Wilcoxon test *p* = 0.02). Segregating for BDNF genotype, no difference was found between Val66Val and Val66Met patients (*p* > 0.05). Sham iTBS did not induce any significant change in total MICARS score and subscales (*p* > 0.05).

### Effect of 10 Sessions of Cerebellar iTBS on BDNF Serum Levels

**Fig. 4:**
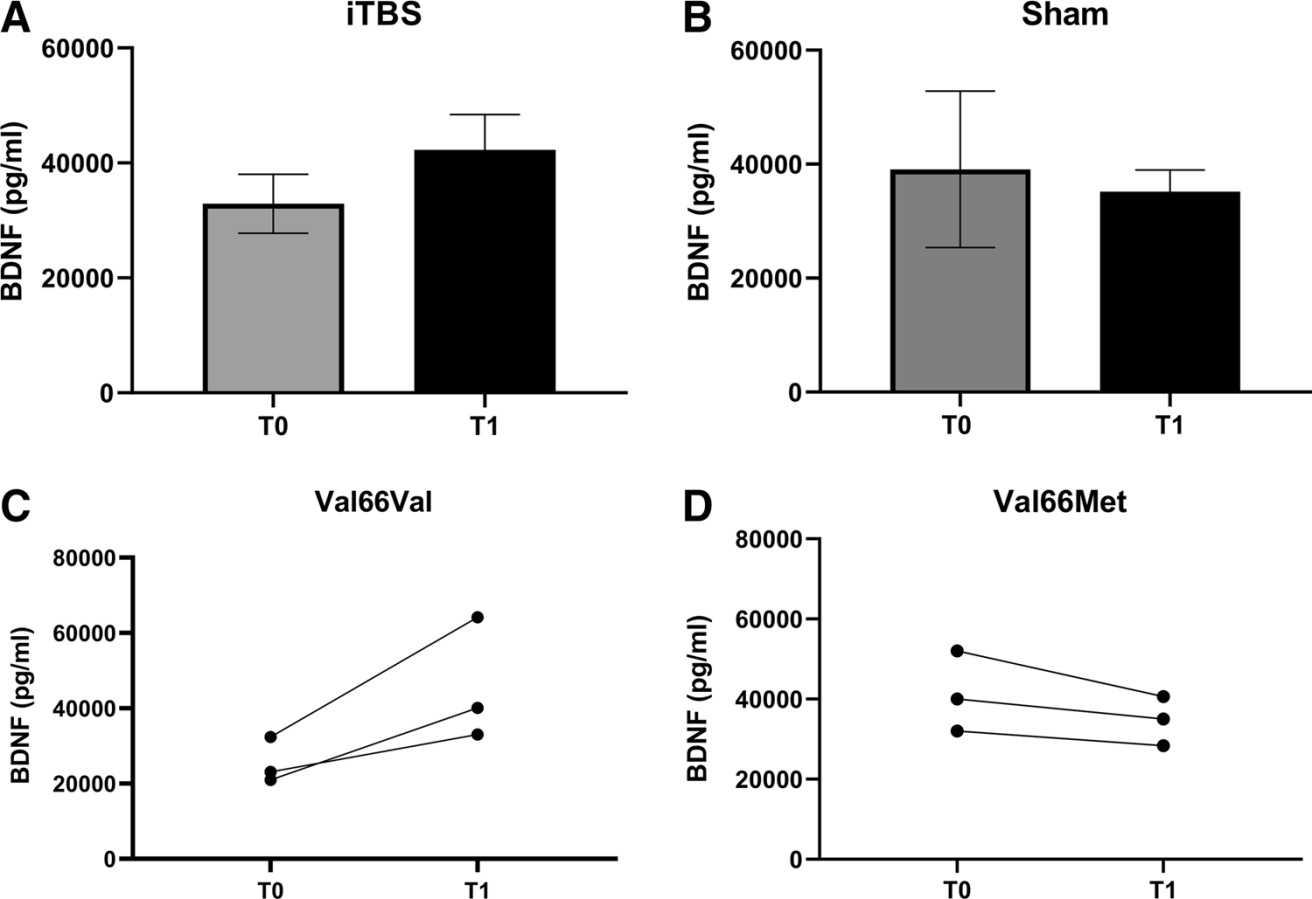
The effect of 10 sessions of cerebellar iTBS (**A**) and Sham stimulation (**B**) on serum BDNF levels. **C**, **D** The effect of cerebellar iTBS on serum BDNF Val66Val and Val66Met patients, respectively

When considering all samples, no significant modification of serum BDNF levels was found in the real iTBS group (Fig. 4A), but segregating for genotype, BDNF was increased in all three Val66Val patients while decreasing in the three Val66Met patients (Fig. 4C and D, respectively). Sham stimulation did not induce any significant change in serum BDNF level (*p* > 0.05) (Fig. 4B) with no apparent influence of Val66Met polymorphism.

## Discussion

Present data show that one session of cerebellar iTBS is able to increase motor cortex excitability in SCA38 patients, demonstrated by an increase of MEP amplitude, comparable with what was previously described for healthy subjects [12]. It has been proposed that cerebellar modulation of motor cortex excitability by iTBS involves the cerebellar-thalamus-cortical (CTC) pathway. CTC projections activated by cerebellar iTBS decrease the activity of GABA(B) inhibitory cortical interneurons, hence increasing MEP amplitude [12]. Patients affected by SCA 38 and other neurodegenerative ataxias show an impairment of CTC projections as revealed by the cerebellar brain inhibition (CBI) TMS protocol [8, 10] which can be improved by different NIBS approaches [10, 36]. Thus, the present data suggest that one session of cerebellar iTBS is able to improve CTC pathway function in SCA38 patients. Moreover, we propose that the observed effect might be considered a predictor of response to therapeutic iTBS in SCA-38, considering that patients with neurodegenerative disorders often show aberrant plasticity of the motor system which limits the effect of different stimulation protocols [4, 17, 37, 38]. The influence of Val66Met BDNF polymorphism on motor cortex excitability in healthy individuals and different models of diseases is an object of debate; indeed, while some authors claim that the polymorphism may affect the response to TBS protocols impairing LTP and LTD mechanisms of synaptic plasticity [16, 23], other authors described no significant effect [39–41]. In this clinical setting, no influence of Val66Met polymorphism was found on motor cortex excitability after a single session of cerebellar iTBS, with all patients displaying a comparable increase in MEPs.

This report shows a significant effect of cerebellar iTBS in improving motor symptoms in SCA-38 patients. The main effect was seen in the posture and gait subscores of MICARS, in agreement with previous studies displaying an improvement in the same items after cerebellar stimulation in other neurodegenerative ataxias [10, 42] and other clinical settings [35, 43]. Possibly, increasing the length of the treatment and the number of patients could eventually lead to an amelioration of other clinical features, such as kinetic functions and dysarthria, which has shown a non-significant tendency to improve.

This study also showed that, although cerebellar iTBS did not induce a significant change on serum BDNF when considering the whole group, segregating for genotype serum BDNF increased in Val66Val and decreased in Val66Met patients. BDNF is recognized as a major player in the long-term effect of rTMS [30] and has been involved in the pathogenesis of SCAs [44] and several degenerative disorders [19, 20]; thus, changes in serum BDNF have been considered a candidate biomarker of efficacy. Despite this evidence, BDNF serum levels are differently modulated by rTMS protocol in healthy individuals and different neurological disorders. A recent meta-analysis [28] suggests that these conflicting results might be influenced by the impaired brain plasticity occurring in degenerative disorders as well as by the presence of Val66Met polymorphism. The present results might be seen accordingly with this hypothesis, but, due to the small number of patients, we cannot make any conclusive statement.

## Conclusion

The main limit of this study is the small size of the sample which lowers the significance of the results especially regarding BDNF data. Indeed, SCA38 is a rare disease affecting a very small number of families living in distant areas, which limits the possibility to include more patients.

Another limit is the lack of a sham iTBS treatment and a control group when probing motor cortex excitability in SCA38 patients. Nevertheless, presented data are very reliable, showing high significance and low variability among patients.

Despite these limits, the effect of cerebellar iTBS in modulating motor cortex excitability and in improving motor symptoms was reliable and consistent with previous studies performed in other types of ataxia. Overall, this exploratory study helps to elucidate the physiopathology of SCA38 and confirms the potential role of cerebellar iTBS in improving motor symptoms of spinocerebellar ataxias which often display limited therapeutic options.
